# Rhodotorula Yeast Culture Improved the Antioxidant Capacity, Lipid Metabolism, and Immunity of Sheep Livers

**DOI:** 10.3390/vetsci12040314

**Published:** 2025-03-30

**Authors:** Xinyu Lu, Huiru Ma, Yeqing Liu, Meiru Chen, Jianlong Dang, Xiangtan Su, Yahui Zhao, Ke Wang, Guang Yang, Gaowei Zhang, Xiaorui Li, Aiqin Gao, Yuan Wang

**Affiliations:** 1 College of Animal Science, Inner Mongolia Agricultural University, Hohhot 010018, China; 2 Hetao College, Bayannur 015000, China; 3 Inner Mongolia Herbivorous Livestock Feed Engineering Technology Research Center, Hohhot 010018, China

**Keywords:** *Rhodotorula* yeast culture, sheep, antioxidation, lipid metabolism, fatty acid, immunity

## Abstract

There is currently no research on the application evaluation of *R*. *mucilaginosa* yeast culture (RYC) in animal production. Therefore, this study investigated the effects of RYC on the antioxidant capacity, lipid metabolism, and immunity of sheep livers. Twenty-four 3-month-old Duhan male sheep (36 ± 4 kg) were divided into four groups. The control group received a basal diet, the L group received a basal diet + 10 g/sheep/day RYC, the M group received a basal diet + 20 g/sheep/day RYC, and the H group received a basal diet + 40 g/sheep/day RYC. The trial lasted for 75 days. The results showed that the content of glutathione peroxidase in the livers of sheep in group M was significantly increased by 26.6%, and the content of malondialdehyde was significantly decreased by 38% (*p* < 0.05). Additionally, the serum levels of total cholesterol, triglycerides, high-density lipoprotein cholesterol, and low-density lipoprotein cholesterol significantly decreased; the liver content of C16:0 decreased; and the levels of C18:2n6C and C20:1 increased (*p* < 0.05). Furthermore, the contents of cytokines TNF-α and IFN-γ in sheep livers from the M group were also significantly decreased by 20% and 24.8%, respectively (*p* < 0.05). These findings suggest that supplementation with 20 g/sheep/day RYC can enhance antioxidant capacity, improve lipid metabolism, and reduce inflammation in sheep livers, which is advantageous for farming healthy sheep.

## Simple Summary

The use of feed additives to ensure sheep health has attracted increasing attention under China’s current policy of completely prohibiting the addition of antibiotics to feed. *Rhodotorula* is a kind of fungus that can produce carotenoids. Previous findings showed that *R*. *mucilaginosa* offers great potential as a source of antioxidants. Yeast culture, a type of yeast additive, exhibits beneficial effects for lipid metabolism and immunity. Therefore, we hypothesized that *R*. *mucilaginosa* yeast cultures have the potential to replace feed antibiotics. To test this hypothesis, we explored the effects of *R*. *mucilaginosa* yeast cultures as an additive on the antioxidant capacity, lipid metabolism, and immunity in sheep liver samples. Adding 20 g/day *R*. *mucilaginosa* yeast culture significantly increased the activities of antioxidant enzymes, decreased blood lipid levels, improved fatty acid composition, and decreased the levels of pro-inflammatory factors in sheep livers. These results indicate that *R*. *mucilaginosa* yeast culture contributed to maintaining a healthy sheep herd, providing a scientific basis for using *R*. *mucilaginosa* yeast culture as a feed additive for sheep.

## 1. Introduction

Sheep play an important role in the Chinese husbandry industry as an important source of meat. However, many common adverse factors exist in current sheep-feeding processes, such as a high proportion of concentrate [1], moldy feed [2], and an unsuitable growth environment [3], that negatively impact the health and production performance of sheep. Previously, antibiotics were commonly added to feed to counteract these negative effects or improve production efficiency. However, unregulated use has led to antibiotic residues in animal products and the emergence of drug-resistant strains in livestock and poultry. These resistant strains can colonize consumers, thereby diminishing the efficacy of antibiotics in treating human diseases [4,5]. Considering the health of consumers, China issued an announcement in 2020 calling for a total ban on the addition of antibiotics during feed production [6]. Therefore, safe and effective feed additives are urgently required for the sheep industry.

Yeast culture (YC) is a unique feed additive produced by fermenting live yeast under fixed culture conditions and then drying the entire medium. Its main components are yeast cell walls and various metabolites [7]. Studies on monogastric animals have shown that YC can affect immunity and intestinal microbial ecology [8,9,10] and increase serum antioxidant enzyme activity [11]. Studies on ruminants have shown that YC can significantly affect the antioxidant level, immune factor content, and fatty acid content in the back muscles of sheep [12,13]. Moreover, YC also impacts rumen microbial diversity, which subsequently affects lipid metabolism-related metabolites within the rumen or modulates the overall rumen environment [14,15]. When used in conjunction with glycerol, YC can improve the energy status of dairy cows and the expression of gluconeogenic enzymes in the liver [16]. Meanwhile, Feng et al.’s research indicates that YC also significantly affects the lipid metabolism pathways in fish liver [17].

*Rhodotorula* species, such as *R. mucilaginosa* and *R. minuta,* are widely distributed fungi, which can produce carotenoids, digestive enzymes, β-glucan, vitamins, and other active metabolites [18,19,20]. The results of a recent mouse study showed that *R. mucilaginosa* significantly improved antioxidant capacity and reduced cyclophosphamide-induced immunosuppression and immune organ atrophy [21]. Hu et al. [19] found that *R. mucilaginosa* improved growth performance, enhanced antioxidant capacity, strengthened gastrointestinal digestion, and maintained the intestinal microbial balance in piglets. Sun et al. [22] showed that an *R. mucilaginosa* solid-state fermentation product improved the laying performance and intestinal flora of hens, speculating that the improved performance might have been related to its metabolites, such as β-glucan. Chen et al. [23] found that the hydrolysate of *R. mucilaginosa* enhanced the antioxidant capacity of juvenile Nile tilapia. In general, previous research has shown great potential of *R. mucilaginosa* as an antioxidant agent.

The liver is central for various metabolic activities in the body, maintaining homeostasis and playing important roles in oxidative stress, lipid metabolism, and immunity [24,25,26]. Current studies have shown that YC has certain effects on sheep antioxidants, lipid metabolism, and immunity. *R. mucilaginosa* has the characteristics of producing carotenoids. Therefore, we speculate that the yeast culture made of *R. mucilaginosa* may have the effect of YC and also show the effect of carotenoids. However, there is no study on the effect of *R. mucilaginosa* yeast culture (RYC) on sheep. Therefore, this study attempts to explore the effects of dietary supplementation of RYC on antioxidants, lipid metabolism, and the immunity of sheep livers and provide a tentative exploration for whether RYC can be used as an alternative antibiotic feed additive.

## 2. Materials and Methods

The animal study protocol was approved by the Animal Welfare and Ethics Committee of Inner Mongolia Agricultural University (approval number NND2022110). RYC was provided by the Chinese Academy of Agricultural Sciences Beijing Institute of Animal Husbandry and Veterinary Medicine, using soybean meal as the solid-state fermentation substrate, and inoculated with the liquid fermentation broth of *R. mucilaginosa*.

### 2.1. Experimental Design

Twenty-four 3-month-old male Duhan sheep (36 ± 4 kg) were randomly assigned to four groups, with six sheep in each group. These groups included the control (CON) group (fed a basal diet), L group (fed basal diet + 10 g/sheep/day RYC), M group (fed basal diet + 20 g/sheep/day RYC), and H group (fed basal diet + 40 g/sheep/day RYC). The entire experiment lasted 90 days (15-day acclimatization phase + 75-day trial period). Throughout the study, all sheep were fed twice daily at 8:00 and 18:00 and were provided with ad libitum access to water. During the trial period, each sheep was fed a specific amount of RYC before 8:00 AM daily, followed by uniform feeding of the basal diet. On the last day of the trial period, blood was drawn from all sheep in the morning before they were fed, and serum samples were subsequently obtained. Fasting and water-deprivation for a whole night. On the following day, five sheep were randomly selected from each group for the Islamic method of slaughter [27] and sampling.

### 2.2. Diet Composition

The basic diet used in this experiment was prepared by Fuchuan Feed Co., Ltd. (Inner Mongolia, China) of Inner Mongolia according to the “NY/T 816-2021 Nutritional Requirements for Mutton Sheep in China”. The ratio of concentrate to roughage was 5:5. The nutritional components of the basal diet used in this study are presented in Table 1.

### 2.3. Sampling

Sheep blood was collected from the jugular vein using a disposable vacuum blood-collection vessel. After standing at room temperature for 40 min, each blood sample was centrifuged at 3000 rpm for 10 min, after which the serum was collected and stored at −20 °C. After the sheep were slaughtered, the surface blood stains were washed with cold normal saline. The central portion of the liver from each sheep was then excised using surgical scissors and placed into a cryogenic storage tube (Corning, NY, USA). Following this, the samples were immediately frozen in liquid nitrogen and subsequently transferred to a −80 °C freezer for storage.

### 2.4. Liver-Antioxidant Capacity

Total superoxide dismutase (T-SOD), catalase (CAT), glutathione peroxidase (GPx), total antioxidative capacity (T-AOC), malondialdehyde (MDA), and protein levels were measured using appropriate kits from Nanjing Jiancheng Bioengineering Institute (Nanjing, China), per the manufacturer’s instructions. The liver homogenate for the experiment was prepared according to the instructions.

### 2.5. Serum Lipid Metabolism

Serum glucose (GLU), total cholesterol (TC), triglycerides (TG), high-density lipoprotein cholesterol (HDL-C), and low-density lipoprotein cholesterol (LDL-C) concentrations were determined using appropriate kits from Nanjing Jiancheng Bioengineering Institute (Nanjing, China), per the manufacturer’s instructions. Free fatty acid (FFA) contents were determined using an ELISA kit from Baoman Biological Technology Co., Ltd. (Shanghai, China).

### 2.6. Liver Fatty Acid Content

Livers (0.6 g) were weighed, ground into powder, added to 0.7 mL of 10 mol/L KOH and 5.3 mL of methanol, mixed well, placed in a 55 °C water bath for 1.5 h, with occasional oscillatory mixing during the period, and cooled to room temperature; then, 0.58 mL of 12 mol/L H_2_SO_4_ was added; it was mixed again, mixed in a 55 °C water bath for 1.5 h, with occasional oscillatory mixing during the period, and cooled to room temperature; 3 mL n-hexane was added, it was vortexed for 5 min and centrifuged at 1500× *g* for 5 min. Then, 1 mL supernatant filtered through a 0.22 μm filter membrane was collected by loading a glass bottle. Fatty acid contents were determined using a 6890N gas chromatograph (Agilent Technologies Inc., Santa Clara, CA, USA), according to a previous method described by Zhao [28].

### 2.7. Liver Immune Cytokines

We used appropriate ELISA kits (Baoman Biological Technology Co., Ltd., Shanghai, China) to measure interleukin 1β (IL-1β), interleukin 6 (IL-6), interleukin 10 (IL-10), tumor necrosis factor-α (TNF-α), and interferon-γ (IFN-γ) levels in the homogenates. The liver homogenate for the experiment was prepared according to the instructions. Protein quantification was performed as described in Section 2.4.

### 2.8. Liver RNA Extraction and Quantitative Real-Time PCR

Liver samples (0.05 g) were added to 1 mL of RNAiso Plus reagent (Takara, Dalian, China), and total RNA was extracted per the manufacturer’s instructions. An Implen P330 device (Implen, Munich, Germany) was used to determine the RNA concentrations. Complementary DNA (cDNA) was generated using an ABI Veriti 96 instrument (Applied Biosystems, Waltham, MA, USA) and a reverse transcription kit (AGbio Co., Ltd., Changsha, China). The cDNA was temporarily stored at −20 °C. A qPCR kit (AGbio Co., Ltd., Changsha, China) and LightCycler 480II Instrument (Roche, Basel, Switzerland) were used to detect the mRNA-expression levels of genes related to antioxidant, lipid-metabolism, and immune activities. β-actin expression was detected as an internal reference gene. The relative expression results were calculated using the 2^−ΔΔCt^ method. Primer design was first performed by checking the relevant mRNA sequences using the Primer-BLAST function of the National Center for Biotechnology. The primers were synthesized by Beijing Liuhe BGI Co., Ltd. (Beijing, China). The specific primer sequences are shown in Table 2.

### 2.9. Data Analysis

All data normality and homoscedasticity were verified through Shapiro–Wilk’s and Levene’s test, respectively, and *p* > 0.05 was considered as normally distributed data [29]. Subsequently, a one-way ANOVA was conducted across the four groups [30]. The model used for the one-way ANOVA can be expressed as

Y ij = μ + τ i + £ ij

where Y ij represents the j-th observed value of the i-th treatment group; μ is the overall mean; τ i is the effect of the i-th treatment group; and £ ij is the random error term.

Following the one-way ANOVA, a Duncan’s multiple range test was employed for post hoc comparisons to identify specific differences among the treatment groups. The Duncan test adjusts for multiple comparisons by comparing the means of all groups in a stepwise manner, thereby controlling the family-wise error rate.

All statistical analyses were performed using IBM SPSS Statistics software (Version 21.0; Armonk, NY, USA). *p* < 0.05 was defined as significant difference, and 0.05 ≤ *p* < 0.1 was defined as a trend of difference. The table data are expressed as the mean and standard error of mean (SEM) [31,32], and the picture data are expressed as the mean ± standard deviation (SD). Drawings were made with GraphPad Prism 9.5 (GraphPad, Boston, MA, USA).

## 3. Results

### 3.1. Liver Antioxidant Capacity

Compared with the CON group, the liver T-SOD activity in the H group was significantly increased (*p* < 0.05); the GPx activity in the L, M, and H groups was significantly increased (*p* < 0.05), but there was no significant difference among the three experimental groups. Compared with CON group, the content of MDA in the L, M, and H groups was significantly decreased (*p* < 0.05), but there was no significant difference among the three experimental groups (Table 3).

### 3.2. Expression of Liver Antioxidant-Related Genes

**Figure 1:**
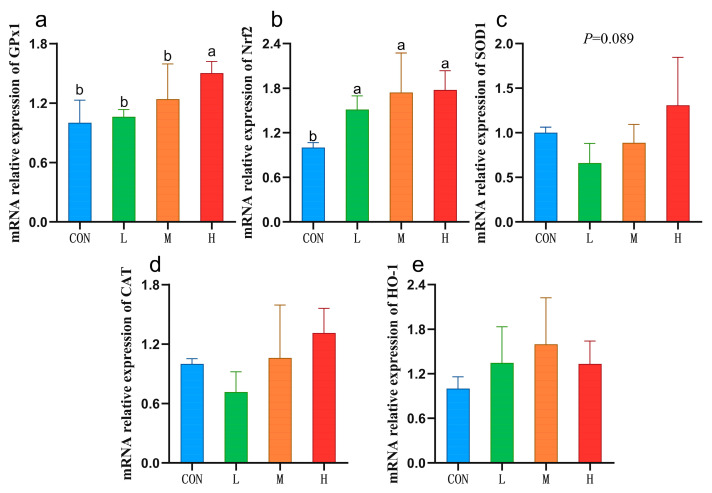
The mRNA expression of antioxidant-related genes in sheep livers. Data are presented as mean ± SD (*n* = 5). Values with different letters indicate statistical significance at *p* < 0.05. CON = fed a basal diet; L = fed a basal diet + 10 g/sheep/day RYC; M = fed a basal diet + 20 g/sheep/day RYC; H = fed a basal diet + 40 g/sheep/day RYC. GPx1: glutathione peroxidase 1; Nrf2: nuclear factor erythroid 2-related factor 2; SOD1: superoxide dismutase 1; CAT: catalase; HO-1: heme oxygenase-1.

Compared with the CON group, the mRNA expression of *GPx1* in the H group was significantly increased (*p* < 0.05), and a trend towards increased *SOD1* mRNA expression was observed (*p* = 0.089). Compared with CON group, the mRNA expression of nuclear factor erythroid 2-related factor 2(*Nrf2*) in the L, M, and H groups was significantly increased (*p* < 0.05), and no significant differences were observed among the three experimental groups (Figure 1).

### 3.3. Serum Lipid Metabolism

Compared with the CON group, the TC content in the L and M groups was significantly decreased (*p* < 0.05); however, no significant difference occurred between the L and M groups. Compared with the CON group, the TG content in the M and H groups was significantly decreased (*p* < 0.05); however, no significant difference occurred between the M and H groups. Compared with the CON group, HDL-C and LDL-C in the L, M, and H groups were significantly decreased (*p* < 0.05), and no significant differences were observed among the three experimental groups (Table 4).

### 3.4. Liver Fatty Acid Profile

Compared with the CON group, we found that the C16:0 contents in livers from the M group significantly decreased (*p <* 0.05), C18:2n6C and C20:1 contents were significantly increased (*p* < 0.05), the C22:2n6 contents of the H group were significantly increased (*p* < 0.05), and the C18:3n6 contents in the H group tended to increase (*p* = 0.05). The ΣPUFAs in the M group was significantly increase (*p* < 0.05; Table 5).

### 3.5. Expression of Liver Lipid Metabolism-Related Genes

**Figure 2:**
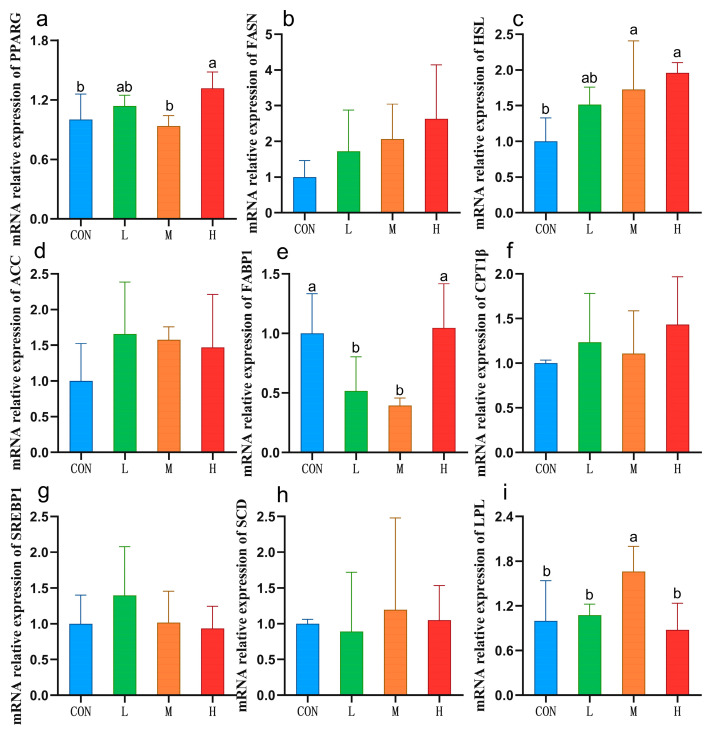
The mRNA expression of lipid metabolism-related genes in sheep livers. Data are presented as mean ± SD (*n* = 5). Values with different letters indicate statistical significance at *p* < 0.05. CON = fed basal diet; L = fed basal diet + 10 g/sheep/day RYC; M = fed basal diet + 20 g/sheep/day RYC; H = fed basal diet + 40 g/sheep/day RYC. PPARG: peroxisome proliferators-activated receptor γ; FASN: fatty acid synthase; HSL: hormone-sensitive lipase; ACC: acetyl CoA carboxylase; FABP1: fatty acid binding protein 1; CPTT1β: carnitine palmitoyltransferase 1β; SREBP1: sterol regulatory element binding protein 1; SCD: stearoyl-CoA Desaturase; LPL: lipoprotein lipase.

Compared with the CON group, the mRNA expression of *PPARG* in the livers from the H group was significantly (*p* < 0.05). In addition, hormone-sensitive lipase (*HSL*) mRNA expression of the M and H groups was significantly increased (*p* < 0.05), fatty acid binding protein 1 (*FABP1*) mRNA expression in the L and M groups was significantly decreased (*p* < 0.05), and the mRNA expression of lipoprotein lipase (*LPL*) in the M group was significantly increased (*p* < 0.05; Figure 2).

### 3.6. Expression of Liver Immune Cytokines and Related Genes

**Figure 3:**
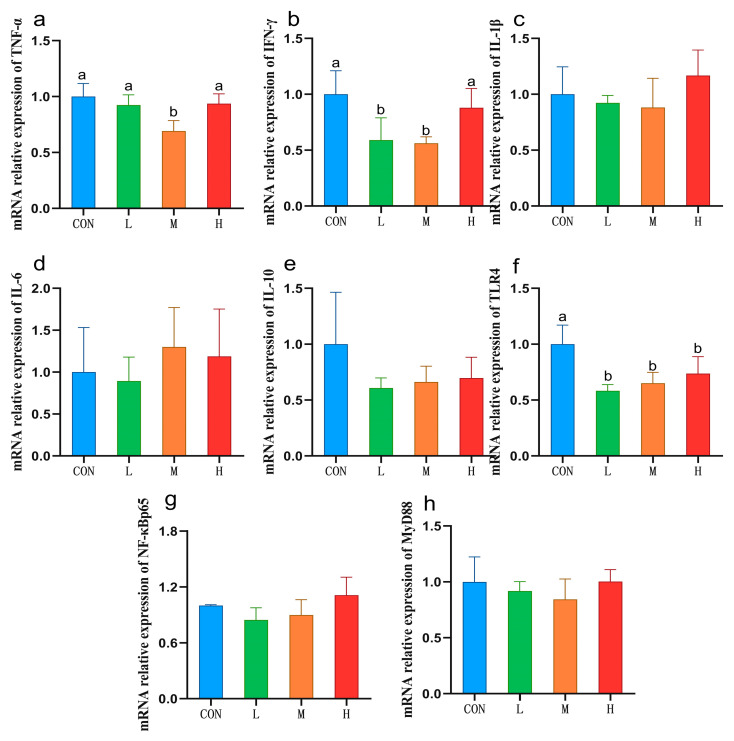
The mRNA expression of immunity-related genes in sheep livers. Data are presented as mean ± SD (*n* = 5). Values with different letters indicate statistical significance at *p* < 0.05. CON = fed basal diet; L = fed basal diet + 10 g/sheep/day RYC; M = fed basal diet + 20 g/sheep/day RYC; H = fed basal diet + 40 g/sheep/day RYC. TNF-α: tumor necrosis factor-α; IFN-γ: interferon-γ; IL-1β: interleukin 1β; IL-6: interleukin 6; IL-10: interleukin 10; TLR4: toll-like receptor 4; NF-κBp65: nuclear factor kappa-B p65; MyD88: myeloid differentiation primary response protein 88.

Compared with the CON group, the contents of TNF-α and IFN-γ in the liver of sheep in the M group decreased significantly (*p* < 0.05; Table 6). Compared with the CON group, the mRNA expression of *TNF-α* in the liver of the M group was significantly decreased (*p* < 0.05), the mRNA expression of *IFN-γ* in the L and M groups was significantly decreased (*p* < 0.05), and there was no significant difference between the two groups. Compared with the CON group, the mRNA expression of *TLR4* in the L, M, and H groups all significantly decreased (*p* < 0.05), and there was no significant difference among the three experimental groups (Figure 3).

## 4. Discussion

Livestock are exposed to various stressors during growth that cause oxidative stress and damage their health. MDA is generally considered the marker of oxidative stress, and the SOD, GPx, and CAT enzymes play important antioxidant roles [33,34]. In this study, all three doses of RYC significantly increased GPx activity and decreased MDA contents in sheep livers, and a daily intake of 40 g RYC significantly increased T-SOD activity. This is consistent with the results of Chen et al. [12]. These findings indicate that RYC can stimulate the antioxidant enzyme system in sheep livers to adapt to ROS attacks and reduce liver oxidative damage. Specifically, RYC can protect cells by increasing GPx activity to remove H_2_O_2_ and increasing T-SOD activity to reduce cell membrane lipid peroxidation [12]. Carotenoids are the main metabolites of *Rhodotorula*, with conjugated double bonds, which can eliminate free radicals in the order of electron transfer, hydrogen extraction, and addition [35], which may be the reason why RYC exerts antioxidant capacity.

Nrf2 is a key regulator of metabolic redox reactions that directly regulate *GPx* expression [36]. Here, we observed that all three doses of RYC tested significantly increased *Nrf2* and *GPx1* mRNA expression (consistent with the trends in their enzyme activities), indicating that RYC increased GPx activity by upregulating the *Nrf2*-*GPx1* signaling pathway. We also found that *SOD1* mRNA expression tended to be elevated in sheep given 40 g/day RYC, whereas the SOD enzyme activity increased significantly. There may be two reasons for this statistical inconsistency in gene- and protein-expression levels. One is related to the nature of SOD. The activity of T-SOD measured in this experiment is the sum of SOD1 and SOD2 isoenzymes. RYC may increase the expression of both isoenzyme genes and eventually lead to an increase in total enzyme activity. The second is the cascade amplification of protein translation, which makes SOD significantly increase at the protein level.

The health status of domestic animals can be reflected by specific blood indicators. In ruminants, the source of GLU mainly depends on endogenous synthesis, particularly the gluconeogenesis pathway [37]. Therefore, GLU levels often reflect the energy level and health status of animals, influenced by their diet and foraging behaviors for a period of time. In this study, we found no differences in GLU levels between the groups, suggesting that the energy intake of sheep in each group was consistent. This finding is also consistent with Ge et al. [16]; they found that YC (*S. cerevisiae*) supplementation did not affect the blood GLU content of dairy cows before and after calving. This indicates that, akin to *S. cerevisiae* YC, RYC as a feed additive does not influence the energy status of sheep. In this experiment, the energy intake of sheep in each group was consistent. TC is mainly synthesized in the liver of animals and depends on different lipoproteins for transport in the body to form LDL-C and HDL-C [38]. Goldstein et al. [39] demonstrated that the TC concentration in the blood correlated positively with low-density lipoprotein levels. The results of that study’s serum TC contents were markedly lower in the L and M groups than in the CON group. Additionally, the LDL-C contents in the L, M, and H groups decreased by 40.5%, 32.4%, and 29.7%, respectively, and the HDL-C contents decreased by 14.9%, 19%, and 16.5% in the same groups. These findings indicate that RYC can decrease TC, LDL-C, and HDL-C levels in sheep, with a greater reduction in LDL-C than in HDL-C, which is beneficial to sheep health. This effect may be related to the presence of β-glucan in RYC [40].

TGs are esterified by one molecule of glycerol and three molecules of fatty acids, which can be hydrolyzed into FFAs to generate energy for the body [41]. However, elevated FFA blood levels can lead to ectopic lipid deposition, which adversely affects animal health [42]. FFA levels reliably reflect the nutritional status and current response levels of sheep to environmental stresses [37]. Carpinelli et al. [43] found that YC reduced the FFA content in the blood of perinatal cows. Malekkhahi et al. [44] found that YC reduced the TG content in the blood of Baluchi lambs fed a high level of YC. Consistently, in this study, we found that adding 20 g or 40 g RYC to the feed per day significantly reduced the serum TG contents of sheep and tended to reduce the serum FFA contents. It indicated that RYC could improve lipid metabolism and reduce disease risk in sheep, with 20 g RYC/day being the most effective.

Fatty acids in the animal body mainly originate from an exogenous daily diet and endogenous de novo synthesis [45,46]. The main product of de novo synthesis is C16:0, and longer fatty acids need to be synthesized on this basis [47]. However, mammals lack desaturation enzymes involved in the synthesis of C18:2n6 and C18:3n3. These two FAs must be obtained from food. A previous report showed that C16:0 can induce inflammation and cell death in animals [48]. Therefore, the significant reduction of C16:0 in group M observed in this study may suggest a diminished risk of inflammation in sheep. This reduction is likely due to the promotion of the conversion to C18:0, which in turn results in an increasing trend in the content of C18:0.

C20:1 can either be obtained from food or synthesized from C18:1 in the body [49]. As all sheep in this study were fed the same basal diet, the observed changes in the C20:1 level were most likely caused by changes in the sheep’s metabolic processes. In this study, the C20:1 level was significantly higher in the M group than in the CON group, whereas C18:1C levels tended to decrease, indicating that the daily addition of 20 g RYC promoted the transformation of C18:1 to C20:1 in sheep livers. This finding differed slightly from those of Li et al. [50]. In their study, YC significantly increased both C18:1C and C20:1 in sheep muscles. This difference may have been caused by different yeasts used in both studies, different principal components in the YC, different sheep breeds, and different tissue specificities.

C18:2n6C enhances the metabolic adaptability and antitumor immunity of CD8 T cells [51] and can be converted into C18:3n6. C22:2n6 has been found to have anti-inflammatory, anti-tumor, and antioxidant functions [52]. In this study, the C18:2n6C and ∑PUFAs contents were significantly higher in the M group than in the CON group, the C18:3n6 content tended to increase in the H group, and the C22:2n6 content was significantly higher, indicating that the daily addition of 20 g RYC to the feed increases the ability of the liver to absorb C18:2n6C without affecting C18:3n6 formation. To improve the ability of the liver to transform C18:3n6 and uptake C22:2n6, 40 g/day of RYC supplementation is required. Similar to our results, Liu et al. [13] also found that high doses of YC could increase the content of C18:2n6C in sheep longissimus dorsi muscles, indicating that some functional components shared by YC may play a role. Moreover, HRE et al. have shown that carotenoids can also affect polyunsaturated fatty acids (PUFAs) in animal livers [53]. Therefore, it cannot be denied that the effect of RYC on liver fatty acid profiles may be the result of the synergistic effect of various functional substances, but it is not known which components are specific, which is also the problem we will continue to study in the next step. In summary, RYC improved the ability of the liver to take up and synthesize PUFAs, improved the fatty acid composition of the sheep liver, and reduced the risk of inflammation. The effects of adding 20 g/sheep and 40 g/sheep per day were different.

To further explore the mechanism whereby RYC affects lipid metabolism in sheep livers, we conducted a relative quantitative analysis of some key genes. LPL and HSL participate in fat degradation and play roles in catalyzing TG degradation. Previous data have shown that a decrease in LPL activity is the main cause of hypertriglyceridemia [54,55]. The results of this study revealed that *LPL* and *HSL* mRNA expression were significantly higher in the M group than in the CON group, indicating that the daily addition of 20 g RYC may increase *LPL* and *HSL* mRNA expression in sheep, increase LPL and HSL enzyme activities, catalyze TG degradation, and reduce serum TG contents. Sterol regulatory element binding protein 1 (SREBP1) and its target genes, acetyl CoA carboxylase (ACC) and fatty acid synthase (FASN), are related to fatty acid synthesis [56]. No significant changes were observed in the above three genes or in fatty acids shorter than C16:0 in this study, indicating that RYC did not affect the de novo fatty acid synthesis pathway.

The FABP1 transporter can specifically bind to long-chain fatty acids in the cell membrane and participate in fatty acid transport [57]. In this study, we found that *FABP1* expression in the L and M groups was significantly lower than that in the CON group. Previous findings showed that *FABP1* expression correlated positively with the TG and TC contents [58]. Habashy et al. [59] suggested that *FABP1* downregulation led to lower plasma TG concentrations in experimental chickens. These findings indicate that RYC reduced the serum TG and TC contents in sheep by downregulating hepatic *FABP1* mRNA expression, which may also explain the increased hepatic ∑PUFAs contents in the M group. That is, decreased *FABP1* mRNA expression impeded *FABP1* binding to long-chain fatty acids, resulting in more PUFAs remaining in hepatocytes. Peroxisome proliferator-activated receptor γ (PPARγ) is a regulatory factor involved in lipogenesis and lipid metabolism and has been found to have anti-inflammatory and liver protection effects [60]. In this study, we found that daily supplementation of 40 g RYC increased the expression of *PPARG* in the liver, which was beneficial to the health of sheep.

Inflammatory factors play an important role in immune response. Our findings show that including 20 g/sheep/day RYC in the feed led to significantly decrease IFN-γ content and mRNA expression in sheep livers when compared with the corresponding levels in the CON group. Previous data have shown that *Nrf2* activation can inhibit *IFN-γ* production [61]. As mentioned above, we found that *Nrf2* mRNA expression increased in groups with added RYC, which might indicate that RYC can inhibit *IFN-γ* production by activating *Nrf2* mRNA expression. In this study, including 20 g/sheep/day RYC in the sheep feed significantly reduced their hepatic TNF-α contents. Kim et al. [10] reported similar results for chickens. Meanwhile, Li et al. [62] obtained the opposite results on Simmental cattle; they found that YC increased the content of bovine serum IL-1β, IL-6, and IFN-γ. This discrepancy may stem from the utilization of distinct strains, which could engender variations in the active constituents within the YC. Moreover, disparities in experimental animals, feeding conditions, and the sites of index detection might also contribute to these divergent outcomes. We also found that the expression of *TLR4* and *TNF-α* mRNA in the M group was significantly down-regulated compared with CON, while *MyD88* and *NF-κBp65* were not significantly different. Both *TLR4/NF-κB* and *TLR4/MAPK* signaling pathways can activate the expression of *TNF-α* [63,64]. Therefore, we speculated that RYC may reduce the content and mRNA expression of *TNF-α* in sheep livers by down-regulating the *TLR4/MAPK* signaling pathway, rather than the *NF-κB* signaling pathway.

In this study, 20 g/sheep/day and 40 g/sheep/day RYC both had a positive regulatory effect on sheep. In terms of antioxidant capacity, all three doses of RYC had significant effects on GPx and MDA, and 40 g/sheep/day RYC additionally enhanced SOD activity in sheep livers. In terms of lipid metabolism, medium-dose RYC showed a better effect and regulation effect on more fatty acids. In terms of immunity, only medium-dose RYC showed an enhanced effect. Considering the overall effect and actual feeding cost, we believe that 20 g/sheep/day RYC is the optimal dose choice at present.

## 5. Conclusions

The results of this study showed that RYC improved the antioxidant capacity in sheep livers by increasing the mRNA-expression levels of *Nrf2* and related antioxidant enzymes. RYC reduced the blood lipid levels in sheep and improved the fatty acid composition of the liver by regulating *HSL*, *LPL*, and *FABP1* mRNA expression. Moreover, RYC reduced the hepatic levels of TNF-α and IFN-γ in sheep. Taking into account the overall efficacy and associated costs, a daily dosage of 20 g/sheep of RYC emerges as the optimal choice. All in all, administering 20 g of RYC daily to sheep can significantly enhance the antioxidant capacity of sheep livers, improve lipid metabolism, and boost immunity. RYC holds great potential as a substitute for feed antibiotics and carries significant implications for the realization of antibiotic-free livestock production. In the future, we will further optimize the dosage or consider incorporating RYC into the total mixed ration to achieve the desired effects for industrial application.

## Figures and Tables

**Table 1:** Basal diet composition and nutrient levels (dry matter level).

Items	Content (%)
Ingredients	
Sheep grass	9.21
Cornstalks	32.9
Concentrate supplements	31.57
Whole plant corn silage	26.32
Total	100
Nutrient levels	
Metabolic energy ^1^ (MJ/kg)	8.09
Crude protein (%)	11.95
Ether extract (%)	2.13
Neutral detergent fibers (%)	54.86
Acidic detergent fibers (%)	21.6
Calcium (%)	0.69
Phosphorus (%)	0.25

^1^ Metabolic energy was a calculated value, and the remaining nutrients were measured values.

**Table 2:** Primer sequences.

Genes	Sequence(5′-3′)	Product Length (bp)	Accession Number
*CAT*	F:CCGCGCAGAAACCTGATGT R:AAGTAGCCAAAAGCCCCTGC	196	XM_060400054.1
*SOD1*	F:GGAGACCTGGGCAATGTGAA R:CCTCCAGCGTTTCCAGTCTT	182	NM_001145185.2
*GPx1*	F:CGGGACTACACCCAGATGAAT R:GTTCTTGGCGTTTTCCTGATGC	108	XM_004018462.5
*Nrf2*	F:TGTGGAGGAGTTCAACGAGC R:CGCCGCCATCTTGTTCTTG	103	XM_042246639.2
*HO-1*	F:AGGGACCAGACCTTCACAGG R:GCATAAAGCCCCACAGCAAC	166	XM_027967703.3
*PPARG*	F:CTTGTGAAGGATGCAAGGGTT R:CATGCGCCCAAACCTGATG	176	NM_001100921.1
*FASN*	F:AGTGGTCATTCAGGTGCGTG R:ATGACGTAGCTCTTGTGGGC	114	XM_027974304.3
*HSL*	F:TCGCCTTTGAAATGCCTCTGACC R:GCTCCTTGCTGTTCTGTCCTTCC	138	NM_001128154.1
*LPL*	F:CCCGGCTTTGATATTGGGAAG R:CTTTGCCAAGTTTCAGCCAGA	171	NM_001009394.1
*ACC*	F:GTGGTGTGAGATCCTGTGCT R:TTAACGAGTCGCAGTTCGGT	93	NM_001009256.1
*CPT1β*	F:AGCAAACCTTAGCTGTGCCA R:GCGAATCAGGCGTTTCTTCC	168	NM_001009259.1
*SREBP1*	F:GACTGCACGTTCGAAGACAT R:CTCATCGTGGAAGGAGGTGG	164	XM_027974786.2
*SCD*	F:ATGGCGTTCCAGAATGACG R:AAAAGCCACGTCGGGAATTG	103	NM_001009254.1
*ACOX1*	F:CTTGCTGAATCAGGGCACCA R:TCGAAGATGAGTTCCGTGGC	115	XM_060395846.1
*IL-1β*	F:TCCTCCGATGAGCTTCTGTG R:GGAGAGCCTTCAGCACACAT	112	NM_001009465.2
*IL-6*	F:ATCGCAGGTCTAATAACCACTCCAG R:GCAGGAAATTCTCAAGGCTTCTCAG	124	NM_001009392.1
*IL-10*	F:GGGTGTCTACAAAGCCATGAGTGAG R:AGGTTTATGTCGGGGAGTCTAGTCG	143	XM_060395938.1
*TNF-α*	F:ACCTGGACTATGCCGAGTCT R:GAAGGGGATGAGGAGGGTCT	127	NM_001024860.1
*IFN-γ*	F:AAGTTCTTGAACGGCAGCTCTGAG R:TGAGGTTAGATTTTGGCGACAGGTC	142	NM_001009803.1
*TLR4*	F:TGGGTGCGGAATGAACTGGTAAAG R:CTGGATGATATTGGCGGCGATGG	114	NM_001135930.1
*MyD88*	F:ATGGTGGTGGTTGTCTCTGAC R:GGAACTCTTTCTTCATTGGCTTGT	139	NM_001166183.1
*NF* *-* *κBp65*	F:TCTGGCCCCTATGTGGAGAT R:CCCGTGTAGCCATTGATCTTG	155	XM_027959295.2
*β-actin*	F:CCCTGGAGAAGAGCTACGAG R:GGTAGTTTCGTGAATGCCGC	131	NM_001009784.3

**Table 3:** Effects of RYC on antioxidant capacity of sheep livers.

Items	CON	L	M	H	SEM	*p*-Value
T-SOD (U/mgprot)	196.97 ^b^	205.09 ^b^	196.26 ^b^	223.31 ^a^	3.745	0.007
CAT (U/mgprot)	36.47	41.79	40.27	37.94	1.086	0.361
GPx (U/mgprot)	10.45 ^b^	14.62 ^a^	13.23 ^a^	13.74 ^a^	0.573	0.024
T-AOC (μmol/gprot)	72.48	71.50	74.26	73.79	1.311	0.911
MDA (nmol/mgprot)	1.26 ^a^	0.74 ^b^	0.78 ^b^	0.88 ^b^	0.062	0.022

Different letter superscripts in the same row indicate significant differences (*n* = 5). CON = fed basal diet; L = fed basal diet + 10 g/sheep/day RYC; M = fed basal diet + 20 g/sheep/day RYC; H = fed basal diet + 40 g/sheep/day RYC. T-SOD: total superoxide dismutase; CAT: catalase; GPx: glutathione peroxidase; T-AOC: total antioxidative capacity; MDA: malondialdehyde.

**Table 4:** Effects of RYC on serum lipid metabolism in sheep.

Items	CON	L	M	H	SEM	*p*-Value
GLU (mmol/L)	3.41	3.20	3.34	3.34	0.076	0.841
TC (mmol/L)	3.06 ^a^	2.46 ^b^	2.47 ^b^	2.66 ^ab^	0.095	0.040
TG (mmol/L)	0.34 ^a^	0.31 ^ab^	0.22 ^c^	0.25 ^bc^	0.011	0.014
HDL-C (mmol/L)	1.21 ^a^	1.03 ^b^	0.98 ^b^	1.01 ^b^	0.026	0.001
LDL-C (mmol/L)	0.37 ^a^	0.22 ^b^	0.25 ^b^	0.26 ^b^	0.017	0.013
FFA (mmol/L)	0.57	0.56	0.56	0.53	0.005	0.056

Different letter superscripts in the same row indicate significant differences (*n* = 6). CON = fed basal diet; L = fed basal diet + 10 g/sheep/day RYC; M = fed basal diet + 20 g/sheep/day RYC; H = fed basal diet + 40 g/sheep/day RYC. GLU: glucose; TC: total cholesterol; TG: triglycerides; HDL-C: high-density lipoprotein cholesterol; LDL-C: low-density lipoprotein cholesterol; FFA: free fatty acid.

**Table 5:** Effects of RYC on the fatty acid profile of sheep livers (of total fatty acids,%).

Items	CON	L	M	H	SEM	*p*-Value
C8:0	0.04	0.04	0.04	0.04	0.001	0.870
C10:0	0.09	0.09	0.09	0.09	0.002	0.752
C12:0	0.23	0.24	0.23	0.25	0.012	0.963
C14:0	1.31	1.34	1.09	1.39	0.073	0.523
C14:1	0.46	0.44	0.43	0.41	0.014	0.680
C15:0	0.34	0.34	0.32	0.33	0.006	0.889
C16:0	23.43 ^a^	23.28 ^a^	21.60 ^b^	23.82 ^a^	0.310	0.038
C16:1	1.43	1.44	1.22	1.61	0.064	0.209
C17:0	0.76	0.81	0.81	0.80	0.012	0.441
C17:1	0.56	0.65	0.59	0.61	0.014	0.157
C18:0	24.53	25.61	26.57	23.94	0.405	0.089
C18:1C	27.82	25.52	23.28	26.56	0.623	0.052
C18:2n6C	12.49 ^b^	13.02 ^b^	16.02 ^a^	13.27 ^b^	0.408	0.002
C18:3n3	0.26	0.27	0.28	0.30	0.008	0.336
C18:3n6	0.35	0.36	0.34	0.41	0.010	0.050
C20:1	0.26 ^b^	0.27 ^b^	0.32 ^a^	0.25 ^b^	0.009	0.018
C20:3n3	4.11	4.50	4.73	4.34	0.128	0.409
C20:3n6	0.40	0.43	0.44	0.41	0.020	0.888
C22:2n6	0.21 ^b^	0.23 ^b^	0.24 ^ab^	0.26 ^a^	0.006	0.013
C22:6n3	0.91	1.11	1.08	1.05	0.050	0.545
ΣSFAs	50.72	51.75	50.76	50.67	0.264	0.435
ΣMUFAs	30.52	28.31	25.84	29.44	0.663	0.061
ΣPUFAs	18.73 ^b^	19.92 ^b^	23.14 ^a^	20.04 ^b^	0.521	0.008

Different letter superscripts in the same row indicate significant differences (*n* = 5). CON = fed basal diet; L = fed basal diet + 10 g/sheep/day RYC; M = fed basal diet + 20 g/sheep/day RYC; H = fed basal diet + 40 g/sheep/day RYC. ΣSFAs = C8:0 + C10:0 + C12:0 + C14:0 + C15:0 + C16:0 + C17:0 + C18:0; ΣMUFAs = C14:1 + C16:1 + C17:1 + C18:1C + C20:1; ΣPUFAs = C18:2n6C + C18:3n3 + C18:3n6 + C20:3n3 + C20:3n6 + C22:2n6 + C22:6n3.

**Table 6:** Effects of RYC on immune cytokines in sheep livers.

Items	CON	L	M	H	SEM	*p*-Value
IL-1β (pg/mg)	6.91	6.01	6.48	6.74	0.230	0.568
IL-6 (pg/mg)	17.61	15.21	15.70	16.55	0.487	0.343
IL-10 (pg/mg)	2.02	2.06	1.83	2.06	0.052	0.377
TNF-α (pg/mg)	11.01 ^a^	9.68 ^ab^	8.81 ^b^	10.25 ^ab^	0.294	0.041
IFN-γ (pg/mg)	60.68 ^a^	53.13 ^ab^	45.65 ^b^	56.42 ^a^	1.777	0.009

Different letter superscripts in the same row indicate significant differences (*n* = 5). CON = fed basal diet; L = fed basal diet + 10 g/sheep/day RYC; M = fed basal diet + 20 g/sheep/day RYC; H = fed basal diet + 40 g/sheep/day RYC. IL-1β: interleukin 1β; IL-6: interleukin 6; IL-10: interleukin 10; TNF-α: tumor necrosis factor-α; IFN-γ: interferon-γ.

## Data Availability

The original contributions presented in this study are included in the article material.
